# Digital Device Exposure and Cognition Levels of Children in Low- and Middle-Income Countries: Cross-sectional Study in Cambodia

**DOI:** 10.2196/31206

**Published:** 2022-08-31

**Authors:** Hye Hyeon Kim, JooHyun Lee, Ho Heon Kim, Sangho Hwang, Ilcheong Yi, Sambath Kao, DooRa Kim, Hyuk-Sang Sohn, Joohye Kim, Yejin Choi, Sangchul Yoon, Yu Rang Park

**Affiliations:** 1 Department of Biomedical Systems Informatics Yonsei University College of Medicine Seoul Republic of Korea; 2 United Nations Research Institute for Social Development Geneva Switzerland; 3 Department of Malnutrition and Neurology National Pediatric Hospital Phnom Penh Cambodia; 4 DoBrain Inc Seoul Republic of Korea; 5 Graduate School of Public Policy & Civic Engagement Kyung Hee University Seoul Republic of Korea; 6 Department of Special Education Baekseok University Cheonan Republic of Korea; 7 Department of Medical Humanities and Social Sciences Yonsei University College of Medicine Seoul Republic of Korea

**Keywords:** low- and middle-income countries, digital device exposure, children, cognitive function, socioeconomic status

## Abstract

**Background:**

Policy makers and practitioners in low- and middle-income countries (LMICs) are increasingly focusing on the effectiveness of digital devices in the delivery of medical and educational services to children under resource constraints. It is widely known that digital literacy can be fostered through exposure to and education regarding digital devices, which can improve children’s academic performance as well as their search and communication skills in the digital era. However, the correlation between the cognitive function of children and exposure and intensity of the exposure to digital devices has rarely been studied, and the association between digital device exposure and the socioeconomic characteristics and cognitive development of children in LMICs is unknown.

**Objective:**

This study examines the association among exposure to digital devices, socioeconomic status, and cognitive function in children aged 3 to 9 years in Cambodia.

**Methods:**

We used a survey of 232 children that gathered data on familiarity with digital devices, demographic characteristics, and socioeconomic status, as well as a Cambridge Neuropsychological Test Automated Battery test for cognitive function, to examine the association between possible barriers and factors that may influence the cognitive function of children in 2 Cambodian schools from April 22, 2019, to May 4, 2019. A comparative analysis was performed with and without digital exposure, and an association analysis was performed among the variables from the survey and cognitive function.

**Results:**

Significant differences were observed in demographic and socioeconomic characteristics such as school location, family type, and family income according to digital device exposure. The results of the Cambridge Neuropsychological Test Automated Battery tests, except for 1 test related to executive function, indicated no significant differences (*P*>.05) between group A and group B or among the 4 subgroups. Pretest digital device experience and amount of time spent using digital devices during the test had no significant impacts on the cognitive development of the children. Conversely, the multivariate analyses showed that cognitive function was associated with educational expenses per child, school (location), family type, and family income.

**Conclusions:**

These results provide evidence to policy makers and practitioners on the importance of improving socioeconomic conditions, leading to investment in education by implementing programs for children’s cognitive development through digital devices in LMICs.

## Introduction

### Background

Resource-constrained health systems in low- and middle-income countries (LMICs) are considered a key obstacle to achieving sustainable development goals (SDGs), specifically SDG 3, to “ensure healthy lives and promote well-being for all at all ages,” and SDG 4, to “ensure inclusive and equitable quality education and promote lifelong learning opportunities for all” [1-3]. As LMICs are experiencing an unprecedented increase in the number and use of digital devices such as mobile phones, digital health and education initiatives that capitalize on the widespread use of these devices are emerging [4,5]. A digital device is a physical piece of equipment that uses digital data in some way, such as sending, receiving, storing, or processing data [6]. Despite controversies over various digital initiatives and programs using digital devices, such as “One Laptop Per Child,” policy makers and practitioners are still interested in the potential of digital technology to address issues such as the digital divide, the impact of resource constraints on cognitive development, and developmental disabilities in children [7-11]. However, will digital device exposure and intensity of the exposure affect cognitive development in children in LMICs? This question is difficult to answer because most of the research targets high-income countries, adolescents, and adults [12-14].

In addition to foundational skills such as literacy and numeracy, digital literacy and skills are also key to implementing the SDGs in this digital age [2]. Therefore, many have suggested using digital devices for children’s cognitive development to improve information processing, communication skills, and educational attainment [10,15]. Studies have demonstrated that digital devices provide only short-term improvements in children’s cognitive abilities during interventions [11,16-19]. A recent study on patients with an intellectual disability diagnosis showed significant improvement in cognitive function using digital devices [20]. Moreover, most studies have focused on the experience of high-income countries or different age groups and patient-only analyses [14,20-23]. The evidence provided by these studies may not be relevant to LMICs because of their different socioeconomic environments and cultures. Although socioeconomic characteristics of households, both within and without, are known to significantly affect cognitive development, few studies on digital device–using interventions, including on the impact of various levels of digital device exposure on children’s cognitive development, have been conducted in LMICs [24].

### This Study

This study addressed the following questions through a survey and cognitive function tests of children in LMICs:

1. Do demographic and socioeconomic characteristics differ according to digital device exposure in LMICs?
2. Do digital device exposure and intensity of the exposure affect cognitive function in children in LMICs?
3. Is there an association among digital device exposure, demographic and socioeconomic characteristics, and cognitive function of children in LMICs?

## Methods

### Study Design

This cross-sectional study was designed to identify and analyze the correlation between cognitive function and exposure to digital devices in children aged 3 to 9 years at 2 schools in Cambodia. The study focused on the following urban and rural regions in Cambodia to consider the effects of various conditions such as average literacy rates, wealth and income distribution, and educational environments: Sisophon in Banteay Meanchey (rural population: 24%-73%) and Sangkat Chaom Chaov in Phnom Penh (rural population: 6%-40%). Sisophon has a relatively small share of the nationally estimated high-wealth quintile (22.9%) and a lower average of schooling years than Sangkat Chaom Chaov (high-wealth quintile: 84.4%). The schools selected were Xavier Jesuit School (rural) and Mirero School (urban). Xavier Jesuit School has both kindergarten and elementary classes. In total, 4 classes—2 kindergarten classes, 1 first grade class, and 1 second grade class—were selected. As Mirero School has only elementary classes, we selected 2 classes each from the first and second grades.

### Participant Enrollment

To target children eligible for the cross-sectional study, we selected regions and schools with the advice of the Korea International Cooperation Agency Cambodia Office, which employs experts who are aware of the overall living environment, including education, in Cambodia. After the selection of areas and schools, classrooms and grades of age that met the inclusion criteria were selected.

**Figure 1:**
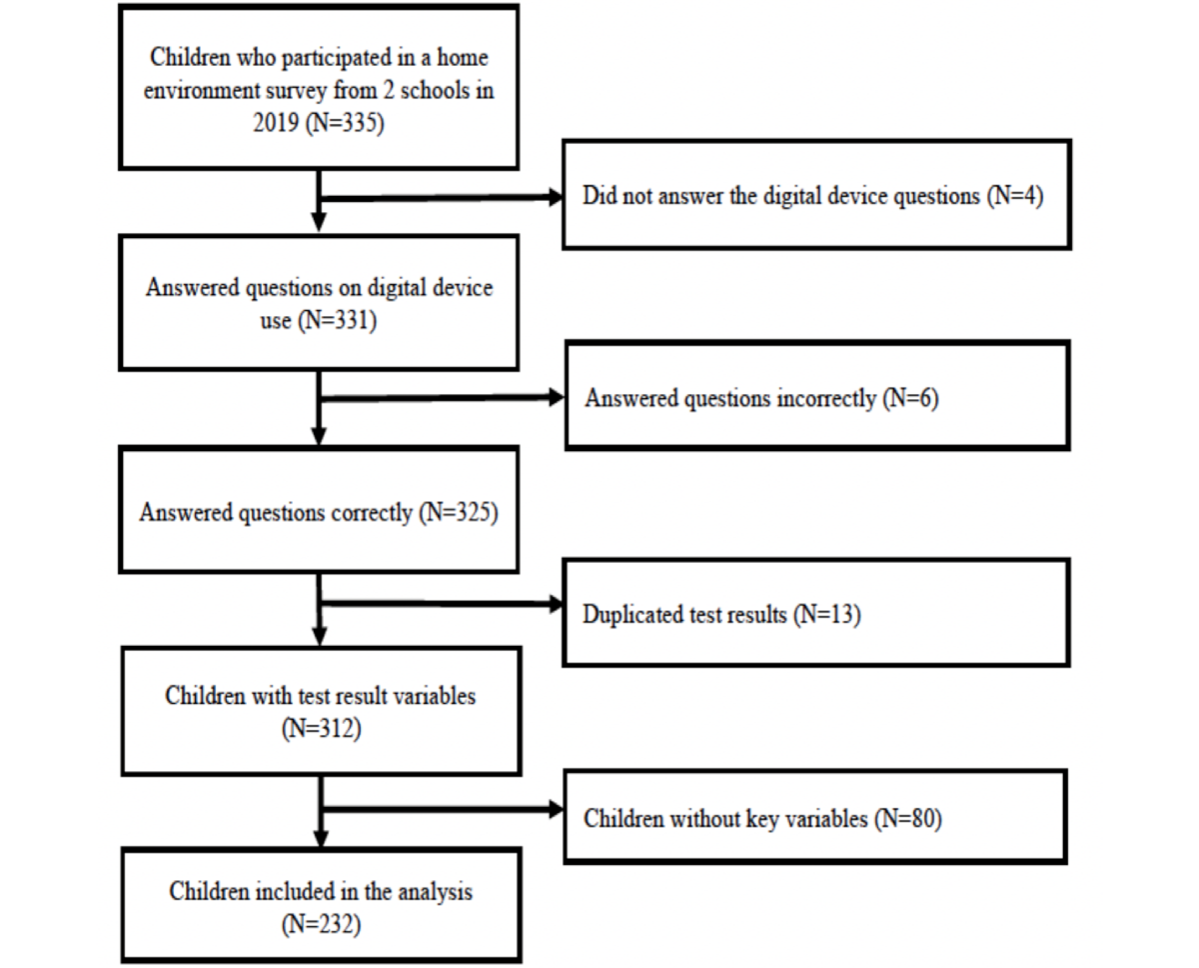
Participant selection flow.

Study participants were enrolled from 2 elementary schools in Cambodia’s 2 regions: rural and urban. The inclusion criteria for children were as follows: (1) male and female students aged 3 to 9 years, (2) no environmental change in their home or school during the study, and (3) children who provided consent from their legal guardian or themselves. Students were excluded from the test if they could not use the study’s digital device because of physical conditions or when legal guardians did not provide consent for the test. Figure 1 shows details of the participant selection flow.

The participants were divided into 2 groups based on their responses to the questionnaire about digital device experience: group A comprised children with digital device experience, and group B comprised those without digital device experience. The participants in group A were subsequently divided into 4 subgroups according to the duration (in minutes) of digital device use: group A-1: <30 minutes per day, group A-2: 30 to 60 minutes per day, group A-3: 60 to 90 minutes per day, and group A-4: >90 minutes per day. Of the 232 participants, 162 (69.8%) were in group A, with 95 (58.6%) in group A-1, 47 (29%) in group A-2, 10 (6.2%) in group A-3, and 10 (6.2%) in group A-4, whereas group B had 70 (30.2%) participants.

### Ethics Approval

All participants provided written informed consent before enrollment in the study. Consent was granted by their guardians after the study was explained in writing. Ethical clearance was obtained from the Ministry of Health’s National Ethics Committee for Health Research in Cambodia (212).

### Data Collection

We gathered data on demographic and socioeconomic characteristics through surveys and data on cognitive function using the Cambridge Neuropsychological Test Automated Battery (CANTAB) test. The survey targeted the basic socioeconomic characteristics and demographics of the participants and their guardians from April 22, 2019, to May 4, 2019. Most of the guardians were either the parents or grandparents. At both schools, the survey was delivered to the guardians living with the participant. The response rate was 82.9%. Most (335/404, 82.9%) of the participants returned the completed form to the school. In cases where the guardians could not read, schoolteachers assisted them in completing the survey at school. The questionnaire had 2 parts. The first elicited demographic information about the children, such as sex, age, and siblings, as well as digital device exposure and use. The second collected information about the family’s socioeconomic status, including residential conditions, guardians’ occupation and education level, household income, and educational expenditure (Multimedia Appendix 1). The participants’ cognitive function, depending on their experience with digital devices, was evaluated through the CANTAB test using a tablet device. The CANTAB test includes highly sensitive, precise, and objective measures of cognitive function correlated with neural networks [25]. It includes tests that evaluate 4 cognitive areas: attention and psychomotor speed, executive function, memory, and social and emotional cognition. The participants were asked to perform 5 tests: motor screening task, reaction time, spatial working memory (SWM), pattern recognition memory, and spatial span. We collected 53 test result variables, including 11 key variables, for measuring the outcome of each test (Table 1 and Multimedia Appendix 2).

**Table 1:** Summary of the Cambridge Neuropsychological Test Automated Battery tests.

Cognitive function and text		Description	Key variable
**Attention and psychomotor speed**			
	Motor screening	To evaluate response speed and pointing accuracy (selecting the cross), participants are asked to select the cross that appears on the screen as quickly and accurately as possible [26]	Mean latency from stimulus (MOTML^a^)
	Reaction time	Assesses simple reaction time and movement during simple and 5-choice reaction time trials	Median 5-choice reaction time (RTIFMDRT^b^); median 5-choice movement time (RTIFMDMT^c^)
**Executive function**			
	Spatial working memory	Test to find individual hidden tokens without returning to a box where one has previously been found [27]	Total between errors (SWMBE468^d^); between errors (4, 6, and 8 boxes; SWMBE 4,6, and 8, respectively); strategy score (SWMS^e^)
**Memory**			
	Pattern recognition memory	A 2-choice test of abstract visual pattern recognition memory [28]	Percent correct immediate (PRMPCI^f^); percent correct delayed (PRMPCD^g^)
	Spatial span	Test to recall the order in which a series of boxes was highlighted [28]	Longest successful sequence (SSPFSL^h^)

^a^MOTML: motor screening task mean latency.

^b^RTIFMDRT: reaction time median 5-choice reaction time.

^c^RTIFMDMT: reaction time median 5-choice movement time.

^d^SWMBE468: spatial working memory between errors (4, 6, and 8 boxes).

^e^SWMS: spatial working memory strategy.

^f^PRMPCI: pattern recognition memory percent correct immediate.

^g^PRMPCD: pattern recognition memory percent correct delayed.

^h^SSPFSL: spatial span forward span length.

### Statistical Analysis

To compare the demographic differences between groups A and B, a chi-square test was performed on categorical variables such as sex, and a Mann-Whitney *U* test was performed on continuous variables such as age. To compare the differences in cognitive function between the 2 groups, normality was tested for the variables using the Shapiro-Wilk test. Variables satisfying normality were examined using a 2-tailed *t* test, and those that did not satisfy normality were compared between the groups using the Mann-Whitney *U* test. For each comparison, the effect size was calculated for the 2 groups according to digital device exposure and for the 4 subgroups according to digital device use time, and the results of the normality test were compared for possible type 1 statistical errors. In comparing the 2 main groups, Cohen *d* was calculated for the *t* tests, and *r* was calculated using the Mann-Whitney *U* test. To compare the effect size among the 4 subgroups, eta squared (η^2^) was calculated for ANOVA, and epsilon squared (ϵ^2^) was used for the Kruskal-Wallis test. The threshold of statistical significance was set at *P*<.05, and an effect size greater than the small size, depending on its type (Cohen *d*≈±0.20: small, *r*≈±0.10: small*,* η^2^≈0.01: small, and ϵ^2^≈0.01: small), was considered significant for 2-tailed *t* tests. To confirm the association among demographic and socioeconomic characteristics, digital device familiarity, and cognitive function in children, univariate regression analysis was performed for 3 cognitive domains and 11 variables flowing from the CANTAB test. In the multivariate linear regression analysis, only variables that were statistically significant (*P*<.05) through univariate regression analysis were selected, and their effect on the cognitive function variable was evaluated with and without adjusting for age and sex. All statistical analyses were conducted using R (version 3.6.3; R Foundation for Statistical Computing) and Python (version 3.7; Python Software Foundation).

## Results

### Participant Flow

A total of 335 children participated in this study. Of these 335 children, 4 (1.2%) who did not answer the questions about digital device exposure and 6 (1.8%) who answered inconsistently (eg, talking about the purpose of the device, not the experience) were excluded from the data analysis; in addition, 13 (3.9%) children with duplicated test results and 80 (23.9%) without values for the key variables of each test were excluded. Ultimately, of the 335 children, 232 (69.3%) were included in the data analysis (Figure 1).

### Overall Population

Of the 232 children, 162 (69.8%) were in group A, and 70 (30.2%) were in group B. The 162 students in group A comprised 110 (67.9%) Xavier Jesuit School students and 52 (32.1%) Mirero School students, a significant difference (*P*<.05). No significant difference between the groups was observed in terms of sex: in group A, 59.3% (96/162) of the participants were male students, whereas in group B, 56% (39/70) were male students (*P*=.72). The mean ages of the participants in group A and group B were 7.3 (SD 1.5) years and 7.6 (SD 1.3) years, respectively, without significant differences (*P*=.15). The proportion of participants with monthly family income of <US $150 was higher in group B (38/70, 54% vs 53/162, 32.7% in group A), with significant differences (*P*=.004). The proportion of participants with monthly family income of >US $350 was higher in group A (25/162, 15.4% vs 4/70, 6% in group B). The proportion of students whose mothers had secondary education and above was far higher in group A (73/162, 45.1% vs 17/70, 24% in group B), with significant differences (*P*=.01). The proportion of families who spent >US $30 per month on education per child was also higher in group A (24/162, 14.8% vs 4/70, 6% in group B), without overall differences between the 2 groups (*P*=.07; Table 2).

**Table 2:** Comparison of participants’ demographic and socioeconomic characteristics according to digital device exposure^a^.

Variable			Group A^b^, n=162	Group B^c^, n=70	Total, N=232	*P* value	
**School, n (%)**							<.001
	Xavier Jesuit (rural)		110 (67.9)	31 (44.3)	141 (60.8)		
	Mirero (urban)		52 (32.1)	39 (55.7)	91 (39.2)		
**Sex, n (%)**							.72
	Female		66 (40.7)	31 (44.3)	97 (41.8)		
	Male		96 (59.3)	39 (55.7)	135 (58.2)		
Age (years), mean (SD)			7.3 (1.5)	7.6 (1.3)	7.4 (1.4)	.15	
**Family type, n (%)**							.005
	Other		11 (6.8)	4 (5.7)	15 (6.5)		
	Only father		3 (1.9)	4 (5.7)	7 (3)		
	Only mother		13 (8)	12 (17.1)	25 (10.8)		
	Parents and grandparents living together		14 (8.6)	11 (15.7)	25 (10.8)		
	Parents living together		121 (74.7)	37 (52.9)	158 (68.1)		
	No response		0 (0)	2 (2.9)	2 (0.9)		
**Family monthly income (US $), n (%)**							.004
		<150	53 (32.7)	8 (54.3)	91 (39.2)		
		150 to 250	47 (29)	21 (30)	68 (29.3)		
		250 to 350	37 (22.8)	6 (8.6)	43 (18.5)		
		350 to 450	10 (6.2)	1 (1.4)	11 (4.7)		
		>450	15 (9.3)	3 (4.3)	18 (7.8)		
**Education expense per child per month (US $), n (%)**							.07
		15	53 (32.7)	38 (54.3)	91 (39.2)		
		15 to 30	87 (53.7)	36 (51.4)	123 (53)		
		>30	24 (14.8)	4 (5.7)	28 (12.1)		
		No response	0 (0)	1 (1.4)	1 (0.4)		

^a^Full table has been presented in Multimedia Appendix 2.

^b^Digital device exposure group.

^c^Digital device nonexposure group.

### Digital Device Exposure

When comparing the results of the CANTAB test between the 2 groups, the SWM between errors (4 boxes; SWMBE4) variable of group A had a median of 2.0 (IQR 1.0-3.0), whereas that of group B had a median of 2.0 (2.0-3.0); there were significant differences (*P*=.01). However, the effect size was small (*P*=.02). The SWM strategy (SWMS) variable of group A had a median of 12.0 (IQR 10.0-58.3), whereas that of group B had a median of 10.0 (IQR 9.0-12.0); there were significant differences (*P*.002). The effect size was small (*P*=.04). There were no significant differences between groups A and B for the other variables. The smaller values for the motor screening task mean latency (MOTML) and reaction time median 5-choice reaction time (RTIFMDRT) variables were positive, but the median values of group A were 15.5 and 21.2 points higher, respectively, than those in group B. Conversely, the reaction time median 5-choice movement time variable for the exposure group was 13.5 points lower, without a significant difference in median values among the other variables (Table 3).

**Table 3:** Comparison of cognitive function according to digital device exposure.

Cognitive function and variable			Group A^a^, n=162, median (IQR)		Group B^b^, n=70, median (IQR)		Total, N=232, median (IQR)		*P* value		Effect size (*r*)
**Attention and psychomotor speed**											
	MOTML^c^ (ms)^d^	781.1 (690.8-928.9)		765.6 (680.5-968.1)		776.1 (685.6-953.7)		.93		–0.004	
	RTIFMDRT^e^ (ms)^d^	517.0 (462.0-578.0)		495.8 (460.0-551.0)		512.0 (461.0-566.0)		.29		0.001	
	RTIFMDMT^f^ (ms)^d^	271.5 (232.0-325.5)		285.0 (238.5-342.5)		275.0 (235.0-330.5)		.11		0.007	
**Memory**											
	PRMPCI^g^ (%)	75.0 (58.3-91.7)		70.8 (50.0-83.3)		75.0 (50.0-91.7)		.52		–0.003	
	PRMPCD^h^ (%)	66.7 (50.0-75.0)		58.3 (50.0-75.0)		66.7 (50.0-75.0)		.08		0.009	
	SSPFSL^i^ (n)	4.0 (3.0-5.0)		4.0 (3.0-5.0)		4.0 (3.0-5.0)		.99		–0.004	
**Executive function**											
	SWMBE468^j^ (n)^d^	24.0 (20.0-28.0)		25.0 (22.0-28.0)		24.0 (20.0-28.0)		.18		0.003	
	SWMBE4^k^ (n)^d^	2.0 (1.0-3.0)		2.0 (2.0-3.0)		2.0 (1.0-3.0)		.01		0.023	
	SWMBE6^l^ (n)^d^	7.0 (5.0-9.0)		7.0 (6.0-9.0)		7.0 (5.0-9.0)		.60		–0.003	
	SWMBE8^m^ (n)^d^	14.5 (12.0-17.0)		15.0 (13.0-17.0)		15.0 (13.0-17.0)		.61		–0.003	
	SWMS^n^ (n)^d^	12.0 (10.0-58.3)		10.0 (9.0-12.0)		11.0 (10.0-50.0)		.002		0.036	

^a^Digital device exposure group.

^b^Digital device nonexposure group.

^c^MOTML: motor screening task mean latency.

^d^Smaller values indicate more positive changes.

^e^RTIFMDRT: reaction time median 5-choice reaction time.

^f^RTIFMDMT: reaction time median 5-choice movement time.

^g^PRMPCI: pattern recognition memory percent correct immediate.

^h^PRMPCD: pattern recognition memory percent correct delayed.

^i^SSPFSL: spatial span forward span length.

^j^SWMBE468: spatial working memory between errors (4, 6, and 8 boxes).

^k^SWMBE4: spatial working memory between errors (4 boxes).

^l^SWMBE6: spatial working memory between errors (6 boxes).

^m^SWMBE8: spatial working memory between errors (8 boxes).

^n^SWMS: spatial working memory strategy.

### Digital Device Use Time

When comparing the differences in cognitive levels based on the duration of digital device use, the results showed no significant differences among the 4 subgroups, with all effect sizes being small. Although there were no significant differences, the median values of the 2 variables measuring attention and psychomotor speed in group A-4 were higher than those in group A-1. Regarding the visual memory variables, there were no significant differences among the 4 subgroups, and the distribution did not show a linear relationship. Regarding the executive function test, as use time increased, the number of errors decreased, leading to positive results. Regarding the SWM test, the distributions of the 4 subgroups were not different (Table 4).

**Table 4:** Comparison of Cambridge Neuropsychological Test Automated Battery test results according to digital device use time.

Cognitive function and variable		Digital device use time						*P* value		Effect size (ε^2^)
		Group A-1^a^, n=95, median (IQR)	Group A-2^b^, n=47, median (IQR)	Group A-3^c^, n=10, median (IQR)	Group A-4^d^, n=10, median (IQR)	Total, N=162, median (IQR)				
**Attention and psychomotor speed**										
	MOTML^e^ (ms)	785.2 (699.3-988.3)	760.5 (660.4-866.8)	835.2 (641.9-909.1)	864.5 (739.8-1065.9)	781.1 (690.8-928.9)	.24		0.143	
	RTIFMDRT^f^ (ms)	510.0 (453.0-569.0)	519.5 (468.8-560.2)	596.5 (478.0-661.0)	530.5 (468.5-607.0)	517.0 (462.0-578.0)	.48		0.070	
	RTIFMDMT^g^ (ms)	278.0 (240.0-333.2)	260.0 (225.2-295.5)	270.5 (243.5-324.0)	257.8 (191.0-418.0)	271.5 (232.0-325.5)	.26		0.037	
**Memory**										
	PRMPCI^h^ (%)	66.7 (50.0-83.3)	83.3 (62.5-91.7)	83.3 (58.3-91.7)	75.0 (66.7-91.7)	75.0 (58.3-91.7)	.21		0.023	
	PRMPCD^i^ (%)	66.7 (50.0-75.0)	66.7 (54.2-75.0)	70.8 (58.3-75.0)	70.8 (58.3-83.3)	66.7 (50.0-75.0)	.35		0.033	
	SSPFSL^j^ (n)	4.0 (3.0- 5.0)	4.0 (3.0- 5.0)	3.5 (3.0- 4.0)	4.0 (4.0- 5.0)	4.0 (3.0- 5.0)	.64		–0.002	
**Executive function**										
	SWMBE468^k^ (n)	24.0 (20.0-28.0)	24.0 (20.0-28.0)	24.5 (21.0-33.0)	21.0 (19.0-28.0)	24.0 (20.0-28.0)	.70		0.000	
	SWMBE4^l^ (n)	2.0 (0.5- 3.0)	2.0 (1.0- 2.0)	2.0 (1.0- 4.0)	1.5 (1.0- 4.0)	2.0 (1.0- 3.0)	.87		0.020	
	SWMBE6^m^ (n)	7.0 (5.0-10.0)	7.0 (5.0- 9.0)	8.0 (6.0-10.0)	6.0 (4.0-10.0)	7.0 (5.0- 9.0)	.66		–0.006	
	SWMBE8^n^ (n)	15.0 (13.0-17.0)	14.0 (12.5-17.5)	15.5 (12.0-19.0)	13.5 (12.0-15.0)	14.5 (12.0-17.0)	.64		–0.004	
	SWMS^o^ (n)	9.0 (8.5-10.0)	10.0 (9.0-11.0)	9.0 (8.0-11.0)	10.0 (9.0-11.0)	10.0 (9.0-11.0)	.07		0.176	

^a^Digital device use time <30 minutes per day.

^b^Digital device use time 30 to 60 minutes per day.

^c^Digital device use time 60 to 90 minutes per day.

^d^Digital device use time >90 minutes per day.

^e^MOTML: motor screening task mean latency.

^f^RTIFMDRT: reaction time median 5-choice reaction time.

^g^RTIFMDMT: reaction time median 5-choice movement time.

^h^PRMPCI: pattern recognition memory percent correct immediate.

^i^PRMPCD: pattern recognition memory percent correct delayed.

^j^SSPFSL: spatial span forward span length.

^k^SWMBE468: spatial working memory between errors (4, 6, and 8 boxes).

^l^SWMBE4: spatial working memory between errors (4 boxes).

^m^SWMBE6: spatial working memory between errors (6 boxes).

^n^SWMBE8: spatial working memory between errors (8 boxes).

^o^SWMS: spatial working memory strategy.

### Cognitive Function and Socioeconomic Status

All results of univariate linear regression analysis to determine the relationship between demographic characteristics and socioeconomic status and CANTAB test score have been presented in Multimedia Appendix 2.

The measures showed that the 3 variables MOTML, RTIFMDMT, and RTIFMDRT from the *Attention and psychomotor speed* cognitive function were significantly associated with *Age* (all *P*<.001), *School* (all *P*<.001), and *Education expense per child* (all *P*<.001) in the univariate model and multivariate model 1. However, in multivariate model 2, adjusted for age and sex, MOTML was significantly associated with *School*, and RTIFMDRT was significantly associated with *Family type* (Table 5).

**Table 5:** Univariate and multivariate regression models for relationship between cognitive function and survey variables.

Cognitive function and variable			Survey variable				Univariate model					Multivariate model 1					Multivariate model 2^a^		
							Coefficients		*P* value		Coefficients			*P* value		Coefficients			*P* value
**Attention and psychomotor speed**																			
	**MOTML^b^**																		
		Age (years)				–*69.718*^c^		*<.001*		–*42.027*			*.001*		–*42.851*			*<.001*	
		**Education expense per child per month (US $; vs less than US $15)**																	
				15 to 30		*131.021*		*<.001*		–59.960			.08		–36.11			.29	
				>30		–5.502		.91		43.200			.08		32.85			.18	
		School (vs Mirero)				*185.077*		*<.001*		*157.79*			*<.001*		*88.74*			*.01*	
	**RTIFMDMT^d^**																		
		Age (years)				–*16.631*		*<.001*		–*11.968*			*.003*		–*11.654*			*.004*	
		**Education expense per child per month (US $; vs less than US $15)**																	
				15 to 30		*41.118*		*<.001*		–20.140			.07		–14.577			.19	
				>30		5.143		.72		*17.669*			*.03*		15.32			.05	
		School (vs Mirero)				*33.564*		*<.001*		*24.352*			*.01*		7.923			.49	
	**RTIFMDRT^e^**																		
		Age				–*40.904*		*<.001*		–*40.238*			*<.001*		–*40.142*			*<.001*	
		**Education expense per child per month (US $; vs less than US $15)**																	
				15 to 30		*61.556*		*<.001*		–20.140			.07		–14.577			.19	
				>30		4.296		.83		*17.669*			*.03*		15.32			.05	
		**Family type (vs Other)**																	
				Only father		–46.919		.29		–50.240			.23		–27.001			.46	
				Only mother		–*77.933*		*.01*		–*64.930*			*.03*		–41.667			.11	
				Parents and grandparents living together		–*62.333*		*.049*		–*74.410*			*.01*		–45.816			.08	
				Parents living together		–*64.801*		*.01*		–*61.050*			*.01*		–*43.347*			*.04*	
		School (vs Mirero)				*68.184*		*<.001*		*69.650*			*<.001*		1.669			.90	
**Memory**																			
	**PRMPCI^f^**																		
		Age (years)				*4.267*		*<.001*		*4.263*			*<.001*		*4.326*			*<.001*	
		**Family type (vs other)**																	
				Only father		16.587		.08		16.911			.07		14.835			.11	
				Only mother		11.778		.09		10.505			.12		8.324			.21	
				Parents and grandparents living together		11.111		.10		12.293			.07		10.379			.12	
				Parents living together		*15.246*		*.01*		*15.054*			*.01*		*13.540*			*.01*	
		School (vs Mirero)				–*6.705*		*.02*		–*6.817*			*.02*		0.521			.88	
	**PRMPCD^g^**																		
		Age (years)				*3.535*		*<.001*		*3.297*			*<.001*		*3.297*			*<.001*	
		**Education expense per child per month (US $; vs less than US $15)**																	
					15 to 30	–*5.389*		*.03*		*6.632*			*.01*		3.686			.17	
					>30	3.122		.39		–1.203			.56		–0.408			.84	
		**Family income per month (US $; vs less than US $150)**																	
				150 to 250		2.497		.37		1.811			.52		1.221			.66	
				250 to 350		5.715		.07		6.012			.06		5.846			.06	
				350 to 450		–5.824		.29		–7.054			.19		–5.608			.29	
				>450		*10.000*		*.03*		*10.095*			*.02*		*11.375*			*.008*	
	**SSPFSL^h^**																		
		Age (years)				*0.226*		*<.001*		*0.226*			*<.001*		*0.225*			*<.001*	
**Executive function**																			
	**SWMBE468^i^**																		
		**Education expense per child per month (US $; vs less than US $15)**																	
				15 to 30		–0.606		.46		–1.438			.11		–1.069			.25	
				>30		–*2.628*		*.03*		–1.276			.06		–*1.397*			*.04*	
		Sex (vs male)				*1.509*		*.048*		–1.582			.04		–1.570			.04	
	**SWMBE4^j^**																		
		Digital device exposure (vs nonexposure group)				–*0.473*		*.02*		–0.389			.06		–0.390			.06	
		**Education expense per child per month (US $; vs less than US $15)**																	
				15 to 30		–*0.504*		*.01*		0.096			.67		0.119			.60	
				>30		–0.286		.33		–0.262			.01		–0.272			.09	
		School (vs Mirero)				–*0.574*		*.002*		–*0.412*			*.04*		–*0.480*			*.04*	
	**SWMBE8^k^**																		
		Age (years)				–*0.372*		*.047*		–*0.379*			*.04*		–*0.379*			*.04*	
		Sex (vs male)				*1.124*		*.04*		–*1.141*			*.03*		–*1.141*			*.03*	
	**SWMS^l^**																		
		Age (years)				–*5.086*		*<.001*		*2.496*			*.046*		*2.540*			*.04*	
		Digital device exposure (vs nonexposure group)				*13.423*		*<.001*		*7.336*			*.03*		*7.190*			*.04*	
		**Family income per month (US $; vs less than US $150)**																	
				150 to 250		–*9.464*		*.03*		–1.301			.72		–1.105			.76	
				250 to 350		–1.479		.76		–4.089			.32		–4.101			.31	
				350 to 450		–6.306		.46		–7.818			.26		–7.365			.29	
				>450		–1.204		.86		–*14.057*			*.01*		–*14.628*			*.009*	
		**Family type (vs other)**																	
				Only father		0.331		.98		0.551			.96		–0.869			.93	
				Only mother		–*17.289*		*.047*		–10.895			.12		–12.283			.08	
				Parents and grandparents living together		–4.302		.62		–9.254			.20		–10.276			.16	
				Parents living together		–7.994		.27		–6.319			.28		–7.223			.22	
		School (vs Mirero)				*32.062*		*<.001*		*32.221*			*<.001*		*37.065*			*<.001*	

^a^Adjusted for age and sex.

^b^MOTML: motor screening task mean latency.

^c^Statistically significant data are shown in italics.

^d^RTIFMDMT: reaction time median 5-choice movement time.

^e^RTIFMDRT: reaction time median 5-choice reaction time.

^f^PRMPCI: pattern recognition memory percent correct immediate.

^g^PRMPCD: pattern recognition memory percent correct delayed.

^h^SSPFSL: spatial span forward span length.

^i^SWMBE468: spatial working memory between errors (4, 6, and 8 boxes).

^j^SWMBE4: spatial working memory between errors (4 boxes).

^k^SWMBE8: spatial working memory between errors (8 boxes).

^l^SWMS: spatial working memory strategy.

The 3 *Memory* cognitive function variables pattern recognition memory percent correct immediate, pattern recognition memory percent correct delayed, and spatial span forward span length were associated with *Age*, *Education expense per child*, *Family income*, *Family type*, and *School*. In the univariate model and multivariate model 1, pattern recognition memory percent correct immediate was significantly associated with *Age* (all *P*<.001), *Family type* (all *P*=.07)*,* and *School* (all *P*<.05). However, in multivariate model 2, it was significantly related only to *Age* (*P*<.001) and *Family type* (*P*=.01). In the univariate model and multivariate model 1, pattern recognition memory percent correct delayed was significantly related to *Age* (all *P*<.001), *Education expense per child* (all *P*<.05)*,* and *Family income* (all *P*<.05). However, in multivariate model 2, it was significantly associated only with *Age* (*P*<.001) and *Family income* (all *P*=.08). In all 3 regression models, spatial span forward span length was strongly related to *Age* (*P*<.001; Table 5).

The variables SWM between errors (4, 6, and 8 boxes), SWMBE4, SWM between errors (8 boxes), and SWMS corresponding to *Executive function* were related to *Age, Digital device exposure, Education expense per child, Family income per month, Family type, Sex*, and *School.* In the univariate model, SWM between errors (4, 6, and 8 boxes) was significantly related to *Education expense per child* (*P*=.03) and *Sex* (*P*=.05). However, in multivariate model 1, it was not associated with any variable. SWMBE4 was significantly associated with *School* (all *P*<.05) in the 3 regression models. Both *Age* (all *P*<.05) and *Sex* (all *P*<.05) had a significant association in the 3 regression analyses of SWM between errors (8 boxes). The SWMS variable showed a significant relationship with the 3 variables *Age* (all *P*<.05), *Digital device exposure* (all *P*<.05), and *School* (all *P*<.001) in all regression analyses (Table 5).

## Discussion

### Comparison With Prior Work

Since 2015, when the 2030 Agenda for Sustainable Development was adopted at the United Nations General Assembly, many initiatives for the application of digital technologies to policies and programs for development, particularly those associated with SDGs 3 and 4, emerged in various countries, particularly LMICs [5,29]. However, there is insufficient evidence on how digital device use affects children’s cognitive improvement in LMICs [8,30,31]. This study focused on urban and rural Cambodian schools to provide a comprehensive perspective on the adoption and use of digital devices among children. We used a cross-sectional study to examine the cognitive level of 3 domains using the CANTAB, focusing on how exposure to digital devices affects cognitive development in elementary school–age children.

### Principal Findings

Our study found significant differences in demographic and socioeconomic characteristics such as school location, family type, and family income according to digital device exposure. We found that children demonstrated neither superiority nor inferiority in cognitive scores on the CANTAB in 3 cognitive domains depending on digital device use. Given the lack of empirical research on the impact of digital device exposure on cognitive development, we offer two key findings that make a significant contribution to policies and programs for the application of digital devices to health care and education: (1) there is no significant association between exposure to digital devices and cognitive development; (2) however, socioeconomic conditions such as school location, family income, family type, and education expenditure are significantly related to cognitive function [31].

### Strengths and Limitations

Several policy implications can be drawn from this study’s findings. The first is the importance of an enabling environment that maximizes the impact of digital devices on cognitive function and development. The evidence demonstrates that socioeconomic factors significantly affect cognitive function and development in infancy, including memory and enforcement functions [24,27]. For example, US studies found that children from higher socioeconomic backgrounds had higher achievements in visuospatial, memory, and executive functions than those from middle or low socioeconomic backgrounds. Similarly, students from higher socioeconomic backgrounds in Brazil were found to have better memory and executive function results [24]. To what extent do socioeconomic conditions affect children’s cognitive function and development in LMICs? The findings of our study demonstrate that these conditions, specifically education expenditure, enhanced cognitive function and development to a greater extent than did children’s exposure to digital devices. Comments such as “we have higher priorities than laptops” expressed by many delegates to the 2005 World Summit on the Information Society, during which the “One Laptop Per Child” devices were introduced, were supported by our findings. Proper investment in education is a much more significant task than purchases of, and exposure to, digital devices, particularly in LMICs.

The second implication concerns the design and setting of digital devices. Many initiatives on the use of digital devices for children’s cognitive function and development are based on a theoretical framework called the “brain’s rearrangement capacity,” which posits that children learn to associate what they see, hear, and know with symbolic characters [31]. Thus, we collected data indicating the symbolic characters that children see and hear on digital devices (Multimedia Appendix 1). Most children with digital devices experience a desire to play games and watch videos, such as those available on YouTube. By playing games and watching videos, children may not learn to associate what they see, hear, or know with symbolic characters. Our finding on the insignificant impacts of exposure to, or use of, digital devices on cognitive development suggests that the design and setting of the devices that children are exposed to should be effective enough to stimulate and accelerate the “brain’s rearrangement capacity.” Giving children access to digital devices with suitable designs and settings is more important than ever amid a pandemic such as the COVID-19 pandemic, during which almost all schools had closed for long periods worldwide [28].

Our study had several limitations. First, the levels of children’s cognitive function and development were measured only during the survey; the long-term impact of digital device use on cognitive function and development was not assessed. This is a typical limitation of cross-sectional studies with a defined time frame. A longitudinal panel study based on groups of children with different socioeconomic backgrounds and levels of exposure to digital devices is needed to measure the long-term effects of digital devices on cognitive function and development. Second, our study was conducted in 2 areas of Cambodia. We focused on both rural and urban areas to examine the impact of differences in socioeconomic levels. However, this did not cover a sufficient area. Nevertheless, considering the lack of studies on the impact of digital device exposure on children’s cognitive function and development in LMICs, particularly those focusing on both digital device exposure and socioeconomic conditions, our study expands the understanding of the enabling environment required for digital device–use initiatives aimed at children’s cognitive function and development.

## Appendix

Multimedia Appendix 1 Basic information survey for the project.

Multimedia Appendix 2 Demographic characteristics and socioeconomic status of eligible participants.

## Abbreviations

CANTAB: Cambridge Neuropsychological Test Automated Battery
LMIC: low- and middle-income country
MOTML: motor screening task mean latency
RTIFMDRT: reaction time median 5-choice reaction time
SDG: sustainable development goal
SWM: spatial working memory
SWMBE4: spatial working memory between errors (4 boxes)
SWMS: spatial working memory strategy

## Data Availability

The data set of anonymized individual participants collected during the study period, along with the data specification, is available upon request from the author with access to the data.

## References

[1] Puchalski Ritchie LM, Khan S, Moore JE, Timmings C, van Lettow M, Vogel JP, Khan DN, Mbaruku G, Mrisho M, Mugerwa K, Uka S, Gülmezoglu AM, Straus SE (2016). Low- and middle-income countries face many common barriers to implementation of maternal health evidence products. J Clin Epidemiol.

[2] Digital inclusion for low-skilled and low-literate people. UNESCO.

[3] O'Donnell O (2007). Access to health care in developing countries: breaking down demand side barriers. Cad Saude Publica.

[4] Dentzer S (2010). E-health's promise for the developing world. Health Aff (Millwood).

[5] Labrique AB, Wadhwani C, Williams KA, Lamptey P, Hesp C, Luk R, Aerts A (2018). Best practices in scaling digital health in low and middle income countries. Global Health.

[6] (2020). The use of digital devices in marketing library products in an inclusively engaged academic library. Handbook of Research on Digital Devices for Inclusivity and Engagement in Libraries.

[7] Kraemer KL, Dedrick J, Sharma P (2009). One laptop per child. Commun ACM.

[8] Warschauer M, Ames M (2010). Can one laptop per child save the world’s poor?. J Int Affairs.

[9] (2016). World Development Report 2016 : Digital Dividends.

[10] United Nation's Children's Fund (UNICEF) (2018). The State of the World's Children 2017 Children in a Digital World.

[11] Cristia J, Ibarrarán P, Cueto S, Santiago A, Severín E (2017). Technology and child development: evidence from the one laptop per child program. Am Econ J Appl Econ.

[12] Danovitch JH (2019). Growing up with Google: how children's understanding and use of internet‐based devices relates to cognitive development. Human Behav and Emerg Tech.

[13] Meherali S, Rahim KA, Campbell S, Lassi ZS (2021). Does digital literacy empower adolescent girls in low- and middle-income countries: a systematic review. Front Public Health.

[14] Oh SS, Kim K, Kim M, Oh J, Chu SH, Choi J (2021). Measurement of digital literacy among older adults: systematic review. J Med Internet Res.

[15] Information literacy competency standards for higher education. American Library Association Institution Repository.

[16] Neisser U, Boodoo G, Bouchard TJ, Boykin AW, Brody N, Ceci SJ, Halpern DF, Loehlin JC, Perloff R, Sternberg RJ, Urbina S (1996). Intelligence: knowns and unknowns. Am Psychol.

[17] Malamud O, Pop-Eleches C (2011). Home computer use and the development of human capital. Q J Econ.

[18] Beuermann DW, Cristia J, Cueto S, Malamud O, Cruz-Aguayo Y (2015). One laptop per child at home: short-term impacts from a randomized experiment in Peru. Am Econ J Appl Econ.

[19] Haskins R (1989). Beyond metaphor: the efficacy of early childhood education. Am Psychol.

[20] Torra Moreno M, Canals Sans J, Colomina Fosch MT (2021). Behavioral and cognitive interventions with digital devices in subjects with intellectual disability: a systematic review. Front Psychiatry.

[21] Di Giacomo D, Ranieri J, Lacasa P (2017). Digital learning as enhanced learning processing? Cognitive evidence for new insight of smart learning. Front Psychol.

[22] Falloon G (2013). Young students using iPads: app design and content influences on their learning pathways. Comput Educ.

[23] Masataka N (2014). Development of reading ability is facilitated by intensive exposure to a digital children's picture book. Front Psychol.

[24] Hackman DA, Farah MJ, Meaney MJ (2010). Socioeconomic status and the brain: mechanistic insights from human and animal research. Nat Rev Neurosci.

[25] CANTAB The most sensitive and validated cognitive research software available. Cambridge Cognition.

[26] Wilmer HH, Sherman LE, Chein JM (2017). Smartphones and cognition: a review of research exploring the links between mobile technology habits and cognitive functioning. Front Psychol.

[27] Noble KG, McCandliss BD, Farah MJ (2007). Socioeconomic gradients predict individual differences in neurocognitive abilities. Dev Sci.

[28] Policy Brief: education during COVID-19 and beyond. United Nations.

[29] The economic impacts of learning losses. OECD.

[30] Chang A, Tilahun L, Breazeal B (2014). Visualisations of data from the literacy tablet reading project in rural Ethiopia. Proceedings of the Electronic Visualisation and the Arts (EVA 2014) (EVA).

[31] (2015). The reading brain, global literacy, and the eradication of poverty. Bread and Brain, Education and Poverty.

