# Correction: Digital Device Exposure and Cognition Levels of Children in Low- and Middle-Income Countries: Cross-sectional Study in Cambodia

**DOI:** 10.2196/44106

**Published:** 2022-12-06

**Authors:** Hye Hyeon Kim, JooHyun Lee, Ho Heon Kim, Sangho Hwang, Ilcheong Yi, Sambath Kao, DooRa Kim, Hyuk-Sang Sohn, Joohye Kim, Yejin Choi, Sangchul Yoon, Yu Rang Park

**Affiliations:** 1 Department of Biomedical Systems Informatics Yonsei University College of Medicine Seoul Republic of Korea; 2 United Nations Research Institute for Social Development Geneva Switzerland; 3 Department of Malnutrition and Neurology National Pediatric Hospital Phnom Penh Cambodia; 4 DoBrain Inc Seoul Republic of Korea; 5 Graduate School of Public Policy & Civic Engagement Kyung Hee University Seoul Republic of Korea; 6 Department of Special Education Baekseok University Cheonan Republic of Korea; 7 Department of Medical Humanities and Social Sciences Yonsei University College of Medicine Seoul Republic of Korea

In “Digital Device Exposure and Cognition Levels of Children in Low- and Middle-Income Countries: Cross-sectional Study in Cambodia (J Med Internet Res 2022;24(8):e31206)” the authors made one correction.

In the originally published article, Ilcheong Yi’s country of organizational affiliation appeared as:

United Nations Research Institute for Social Development, Geneva, Swaziland

It has now been corrected to:

United Nations Research Institute for Social Development, Geneva, Switzerland

The correction will appear in the online version of the paper on the JMIR Publications website on December 6, 2022, together with the publication of this correction notice. Because this was made after submission to PubMed, PubMed Central, and other full-text repositories, the corrected article has also been resubmitted to those repositories.

