# Supplementary material for: Digital Device Exposure and Cognition Levels of Children in Low- and Middle-Income Countries: Cross-sectional Study in Cambodia
Source: J Med Internet Res. 2022 Aug 31;24(8):e31206. doi: 10.2196/31206 (PMC9475408; doi:10.2196/31206)
Supplement: Multimedia Appendix 1 [file jmir_v24i8e31206_app1.docx]

| **Multimedia appendix 1. Basic Information Survey for Project** |
| --- |

**School: ___________________ Grade____________ Student Name: ____________________**

**<Student>**

**1.** What is your child's gender?

| □ | ① Male | □ | ② Female |
| --- | --- | --- | --- |

**2.** How many brother or sister does he/she have?

| □ | Older Brother ______ | □ | Older Sister  ________ | □ | Younger Brother _______ | □ | Younger Sister  ________ |
| --- | --- | --- | --- | --- | --- | --- | --- |

**3.** What is the average length of time (in school or out-of-school) your child is receiving education per week?

| □ | ① less than 10 hours | □ | ② 10 hours – 20 hours | □ | ③ 20 hours – 30 hours | □ | ④ 30 hours  - 40 hours | □ | ⑤ more than  40 hours |
| --- | --- | --- | --- | --- | --- | --- | --- | --- | --- |

**4.** How much is your child's daily digital device usage time (smartphone / tablet PC / computer)?

| □ | ① None | □ | ② Less than 30 min | □ | ③ 30 min  - 60 min | □ | ④ 60 min – 90 min | □ | ⑤ more than  90 min |
| --- | --- | --- | --- | --- | --- | --- | --- | --- | --- |

**5.** What is your child's primary use of digital devices?

| □ | ① No use | □ | ② Game | □ | ③ Youtube | □ | ④ Academic purpose (ex_School Assignmnet) | □ | ⑤ Internet Search |
| --- | --- | --- | --- | --- | --- | --- | --- | --- | --- |

**<Caregiver>**

**4.** What is your gender?

| □ | ① male | □ | ② female |
| --- | --- | --- | --- |

**5.** What is your age?

| □ | ① 20’s | □ | ② 30’s | □ | ③ 40’s | □ | ④ more than 50’s | □ | ⑤ extra |
| --- | --- | --- | --- | --- | --- | --- | --- | --- | --- |

**6. What is the shape of your family?**

| □ | ① Parents living together | □ | ② Parents and grandparents living together | □ | ③only mother |  | ④ only father | □ | ⑤ extra |
| --- | --- | --- | --- | --- | --- | --- | --- | --- | --- |

**7. Where do you live?**

| □ | ① City | □ | ② Country | □ | ③ extra |
| --- | --- | --- | --- | --- | --- |

**8.** Please choose Father’s education level.

| □ | ① No public education experience | □ | ② Elementary school graduate | □ | ③ middle school graduate | □ | ④ high school graduate |
| --- | --- | --- | --- | --- | --- | --- | --- |
| □ | ⑤ college and university graduate | □ | ⑥ More than a master's course | □ | ⑦ extra | □ |  |

**9. Please choose father's occupation.**

| □ | ① Agriculture and fisheries | □ | ② Services (eg, cosmetics, hotels, travel, restaurants, etc.) | □ | ③ Sales (eg,selling clothes, shoes, etc.) | □ | ④ Technical jobs (eg sewing, transportation, construction, mechanics, telecommunications, etc.) |
| --- | --- | --- | --- | --- | --- | --- | --- |
| □ | ⑤  Administrative and managerial positions (eg, administrative and managerial positions of public institutions and companies) | □ | ⑥  Profession (eg researcher, doctor, teacher, etc.) | □ | ⑦ inoccupation | □ | ⑧ extra |

**10.** Please choose mother’s education level.

| □ | ① No public education experience | □ | ② Elementary school graduate | □ | ③ middle school graduate | □ | ④ high school graduate |
| --- | --- | --- | --- | --- | --- | --- | --- |
| □ | ⑤ college and university graduate | □ | ⑥ More than a master's course | □ | ⑦ extra | □ |  |

**11. Please choose mother's occupation.**

| □ | ① Agriculture and fisheries | □ | ② Services (eg, cosmetics, hotels, travel, restaurants, etc.) | □ | ③ Sales (eg,selling clothes, shoes, etc.) | □ | ④ Technical jobs (eg sewing, transportation, construction, mechanics, telecommunications, etc.) |
| --- | --- | --- | --- | --- | --- | --- | --- |
| □ | ⑤ Administrative and managerial positions (eg, administrative and managerial positions of public institutions and companies) | □ | ⑥ Profession (eg researcher, doctor, teacher, etc.) | □ | ⑦ inoccupation | □ | ⑧ extra |

**12.** What is your family's monthly income?

| □ | ① less than $150 | □ | ② $150- 250 | □ | ③ 250 - 350 |  | ④ 350 - 450 | □ | ⑤ more than $450 |
| --- | --- | --- | --- | --- | --- | --- | --- | --- | --- |

**13.** What is the approximate cost of your child's education (per child) per month?

| □ | ① less than 15$ | □ | ② $15-20 | □ | ③ $20-25 |  | ④ $25-30 | □ | ⑤ more than $30 |
| --- | --- | --- | --- | --- | --- | --- | --- | --- | --- |

**14.** What is the average length of time that adults (parents, grandparents, etc.) spend together at home in relation to their child's education (such as reading books, learning texts and maths, telling stories, singing, drawing, and exercising)?

| □ | ① None | □ | ② less than 1 hour | □ | ③ 1 hour   - 2 hours |  | ④ 2 hours  - 3 hours | □ | ⑤ more than 3 hours |
| --- | --- | --- | --- | --- | --- | --- | --- | --- | --- |

Thank you very much for taking the survey.
