# Supplementary material for: Digital Device Exposure and Cognition Levels of Children in Low- and Middle-Income Countries: Cross-sectional Study in Cambodia
Source: J Med Internet Res. 2022 Aug 31;24(8):e31206. doi: 10.2196/31206 (PMC9475408; doi:10.2196/31206)
Supplement: Multimedia Appendix 2 [file jmir_v24i8e31206_app2.docx]

Multimedia appendix 2.

Table1. Demographic characteristics and socioeconomic status of eligible participants

|  | Digital device exposure | | | *P* value |
| --- | --- | --- | --- | --- |
|  | Yes | No | Total |  |
|  | (n=162) | (n=70) | (N=232) |  |
| School |  |  |  | 0.001 |
| - Xavier | 110 (67.9%) | 31 (44.3%) | 141 (60.8%) |  |
| - Mirero | 52 (32.1%) | 39 (55.7%) | 91 (39.2%) |  |
| Sex |  | | | 0.721 |
| - Male | 96 (59.3%) | 39 (55.7%) | 135 (58.2%) |  |
| - Female | 66 (40.7%) | 31 (44.3%) | 97 (41.8%) |  |
| Age | 7.3 ± 1.5 | 7.6 ± 1.3 | 7.4 ± 1.4 | 0.154 |
| DigitalDeviceUagePerDay |  | | | < 0.001 |
| - 30~60 min | 47 (29.0%) | 0 (0.0%) | 47 (20.3%) |  |
| - 60~90 min | 10 (6.2%) | 0 (0.0%) | 10 (4.3%) |  |
| - less than 30min | 95 (58.6%) | 0 (0.0%) | 95 (40.9%) |  |
| - more than 90 min | 10 (6.2%) | 0 (0.0%) | 10 (4.3%) |  |
| - None | 0 (0.0%) | 70 (100.0%) | 70 (30.2%) |  |
| DeviceUsagePurpose |  | | | < 0.001 |
| - Academic purpose | 6 (3.7%) | 0 (0.0%) | 6 (2.6%) |  |
| **- Game** | **36 (22.2%)** | 0 (0.0%) | 36 (15.5%) |  |
| - Game and Youtube | 15 (9.3%) | 0 (0.0%) | 15 (6.5%) |  |
| - Internet Search | 4 (2.5%) | 0 (0.0%) | 4 (1.7%) |  |
| - No Use | 0 (0.0%) | 70 (100.0%) | 70 (30.2%) |  |
| **- Youtube** | **97 (59.9%)** | 0 (0.0%) | 97 (41.8%) |  |
| - Youtube and Academic | 3 (1.9%) | 0 (0.0%) | 3 (1.3%) |  |
| - Youtube and Internet | 1 (0.6%) | 0 (0.0%) | 1 (0.4%) |  |
| FamilyShape |  | | | 0.005 |
| - extra | 11 (6.8%) | 4 (5.7%) | 15 (6.5%) |  |
| - only father | 3 (1.9%) | 4 (5.7%) | 7 (3.0%) |  |
| - only mother | 13 (8.0%) | 12 (17.1%) | 25 (10.8%) |  |
| - Parents and grandparents living together | 14 (8.6%) | 11 (15.7%) | 25 (10.8%) |  |
| - Parents living together | 121 (74.7%) | 37 (52.9%) | 158 (68.1%) |  |
| - Unknown | 0 (0.0%) | 2 (2.9%) | 2 (0.9%) |  |
| Sibling |  | | | 0.861 |
| - No sibling | 26 (16.0%) | 12 (17.1%) | 38 (16.4%) |  |
| - 1 sibling | 64 (39.5%) | 24 (34.3%) | 88 (37.9%) |  |
| - 2 ~ 3 sibling | 55 (34.0%) | 25 (35.7%) | 80 (34.5%) |  |
| - more than 3 sibling | 13 (8.0%) | 8 (11.4%) | 21 (9.1%) |  |
| - Unknown | 4 (2.5%) | 1 (1.4%) | 5 (2.2%) |  |
| FamilyIncome |  | | | 0.004 |
| - less than $150 | 53 (32.7%) | 38 (54.3%) | 91 (39.2%) |  |
| - $150 - 250 | 47 (29.0%) | 21 (30.0%) | 68 (29.3%) |  |
| - $250 - 350 | 37 (22.8%) | 6 (8.6%) | 43 (18.5%) |  |
| - $350 - 450 | 10 (6.2%) | 1 (1.4%) | 11 (4.7%) |  |
| - more than $450 | 15 (9.3%) | 3 (4.3%) | 18 (7.8%) |  |
| - No response | 0 (0.0%) | 1 (1.4%) | 1 (0.4%) |  |
| FatherEducationLevel |  | | | 0.188 |
| - Elementary school graduate | 63 (38.9%) | 25 (35.7%) | 88 (37.9%) |  |
| - Extra or unknown | 10 (6.2%) | 9 (12.9%) | 19 (8.2%) |  |
| - Secondary education and above | 78 (48.1%) | 28 (40.0%) | 106 (45.7%) |  |
| - No public education | 11 (6.8%) | 8 (11.4%) | 19 (8.2%) |  |
| FatherOccupation |  | | | 0.123 |
| - Administrative and managerial positions | 10 (6.2%) | 2 (2.9%) | 12 (5.2%) |  |
| - Agriculture and fisheries | 37 (22.8%) | 8 (11.4%) | 45 (19.4%) |  |
| - Car driver | 4 (2.5%) | 0 (0.0%) | 4 (1.7%) |  |
| - extra | 5 (3.1%) | 7 (10.0%) | 12 (5.2%) |  |
| - Inoccupation | 7 (4.3%) | 4 (5.7%) | 11 (4.7%) |  |
| - Motobike driver | 1 (0.6%) | 0 (0.0%) | 1 (0.4%) |  |
| - Profession | 7 (4.3%) | 2 (2.9%) | 9 (3.9%) |  |
| - Sales | 13 (8.0%) | 6 (8.6%) | 19 (8.2%) |  |
| - Security guard | 1 (0.6%) | 0 (0.0%) | 1 (0.4%) |  |
| - Services | 5 (3.1%) | 3 (4.3%) | 8 (3.4%) |  |
| - Technical jobs | 68 (42.0%) | 32 (45.7%) | 100 (43.1%) |  |
| - Unknown | 4 (2.5%) | 6 (8.6%) | 10 (4.3%) |  |
| MotherEducationLevel |  | | | 0.014 |
| - Elementary school graduate | 68 (42.0%) | 36 (51.4%) | 104 (44.8%) |  |
| - Extra or unknown | 8 (4.9%) | 5 (7.1%) | 13 (5.6%) |  |
| - Secondary education and above | 73 (45.1%) | 17 (24.3%) | 90 (38.8%) |  |
| - No public education | 13 (8.0%) | 12 (17.1%) | 25 (10.8%) |  |
| MotherOccupation |  | | | 0.047 |
| - Administrative and managerial positions | 4 (2.5%) | 0 (0.0%) | 4 (1.7%) |  |
| - Agriculture and fisheries | 20 (12.3%) | 3 (4.3%) | 23 (9.9%) |  |
| - extra | 6 (3.7%) | 8 (11.4%) | 14 (6.0%) |  |
| - Inoccupation | 28 (17.3%) | 20 (28.6%) | 48 (20.7%) |  |
| - Profession | 5 (3.1%) | 1 (1.4%) | 6 (2.6%) |  |
| - Sales | 26 (16.0%) | 7 (10.0%) | 33 (14.2%) |  |
| - Services | 12 (7.4%) | 3 (4.3%) | 15 (6.5%) |  |
| - Technical jobs | 57 (35.2%) | 27 (38.6%) | 84 (36.2%) |  |
| - Unknown | 4 (2.5%) | 1 (1.4%) | 5 (2.2%) |  |
| EduTimePerWeek |  | | | < 0.001 |
| - less than 10 hours | 11 (6.8%) | 20 (28.6%) | 31 (13.4%) |  |
| - 10 hours - 20 hours | 34 (21.0%) | 15 (21.4%) | 49 (21.1%) |  |
| - 20 hours - 30 hours | 83 (51.2%) | 28 (40.0%) | 111 (47.8%) |  |
| - 30 hours - 40 hours | 30 (18.5%) | 5 (7.1%) | 35 (15.1%) |  |
| - more than 40 hours | 4 (2.5%) | 1 (1.4%) | 5 (2.2%) |  |
| - No response | 0 (0.0%) | 1 (1.4%) | 1 (0.4%) |  |
| EducationExpensePerChild |  | | | 0.068 |
| - less than $15 | 51 (31.5%) | 29 (41.4%) | 80 (34.5%) |  |
| - $15 ~ 30 | 87 (53.7%) | 36 (51.4%) | 123 (53.0%) |  |
| - more than $30 | 24 (14.8%) | 4 (5.7%) | 28 (12.1%) |  |
| - No response | 0 (0.0%) | 1 (1.4%) | 1 (0.4%) |  |
| HomeEducationTime |  | | | 0.067 |
| - less than 1 hour | 89 (54.9%) | 28 (40.0%) | 117 (50.4%) |  |
| - more than 1 hour | 37 (22.8%) | 16 (22.9%) | 53 (22.8%) |  |
| - No response or Unknown | 1 (0.6%) | 2 (2.9%) | 3 (1.3%) |  |
| - None | 35 (21.6%) | 24 (34.3%) | 59 (25.4%) |  |

Group A: digital device exposure group; Group B: digital device non-exposure group

Date are n(%), mean(±sd)

Table 2. Univariate regression model for relationship between cognitive function and survey variables

|  | | | Univariate | |
| --- | --- | --- | --- | --- |
| Cognitive function | Variable | Survey variable | Coefficients | *P* value |
| Attention and psychomotor speed | MOTML | Age | **-69.718** | **<0.001** |
|  |  | Gender (vs Male) | -41.570 | 0.178 |
|  |  | Digital device exposure (vs. non-exposure group) | -4.188 | 0.900 |
|  |  | Education expense per child (vs less than $15) |  |  |
|  |  | - $15–30 | **131.021** | **<0.001** |
|  |  | - more than $30 | -5.502 | 0.907 |
|  |  | Family income (vs. less than $150) |  |  |
|  |  | - $150–250 | -33.037 | 0.377 |
|  |  | - $250–350 | -7.787 | 0.857 |
|  |  | - $350–450 | 0.626 | 0.993 |
|  |  | - more than $450 | 78.783 | 0.191 |
|  |  | Family type (vs Extra) |  |  |
|  |  | - Only father | 8.478 | 0.937 |
|  |  | - Only mother | 3.395 | 0.965 |
|  |  | - Parents and grandparents living together | -14.521 | 0.849 |
|  |  | - Parents living together | -7.215 | 0.909 |
|  |  | School (vs. Mirero) | **185.077** | **<0.001** |
|  | RTIFMDMT | Age | **-16.631** | **<0.001** |
|  |  | Gender (vs Male) | 7.437 | 0.435 |
|  |  | Digital device exposure (vs. non-exposure group) | -17.315 | 0.091 |
|  |  | Education expense per child (vs less than $15) | 5.143 | 0.723 |
|  |  | - $15–30 | **41.118** | **<0.001** |
|  |  | - more than $30 | 5.143 | 0.723 |
|  |  | Family income (vs. less than $150) |  |  |
|  |  | - $150–250 | -21.012 | 0.069 |
|  |  | - $250–350 | -4.856 | 0.715 |
|  |  | - $350–450 | -22.545 | 0.326 |
|  |  | - more than $450 | -15.376 | 0.407 |
|  |  | Family type (vs Extra) |  |  |
|  |  | - Only father | -45.400 | 0.168 |
|  |  | - Only mother | -26.500 | 0.259 |
|  |  | - Parents and grandparents living together | -13.120 | 0.576 |
|  |  | - Parents living together | -11.748 | 0.545 |
|  |  | School (vs. Mirero) | **33.564** | **<0.001** |
|  | RTIFMDRT | Age | **-40.904** | **<0.001** |
|  |  | Gender (vs Male) | 4.35 | 0.737 |
|  |  | Digital device exposure (vs. non-exposure group) | 22.239 | 0.11 |
|  |  | Education expense per child (vs less than $15) |  |  |
|  |  | - $15–30 | **61.556** | **<0.001** |
|  |  | - more than $30 | 4.296 | 0.826 |
|  |  | Family income (vs. less than $150) |  |  |
|  |  | - $150–250 | -9.384 | 0.550 |
|  |  | - $250–350 | 10.307 | 0.569 |
|  |  | - $350–450 | -2.609 | 0.933 |
|  |  | - more than $450 | 21.166 | 0.402 |
|  |  | Family type (vs. extra) |  |  |
|  |  | - Only father | -46.919 | 0.289 |
|  |  | - Only mother | **-77.933** | **0.014** |
|  |  | - Parents and grandparents living together | **-62.333** | **0.049** |
|  |  | - Parents living together | **-64.801** | **0.014** |
|  |  | School (vs. Mirero) | **68.184** | **<0.001** |
| Memory | PRMPCI | Age | **4.267** | **<0.001** |
|  |  | Gender (vs Male) | 3.010 | 0.2816 |
|  |  | Digital device exposure (vs. non-exposure group) | 1.875 | 0.5340 |
|  |  | Education expense per child (vs less than $15) |  |  |
|  |  | - $15–30 | -3.795 | 0.210 |
|  |  | - more than $30 | 3.185 | 0.470 |
|  |  | Family income (vs. less than $150) |  |  |
|  |  | - $150–250 | 3.804 | 0.258 |
|  |  | - $250–350 | 4.737 | 0.223 |
|  |  | - $350–450 | 0.050 | 0.994 |
|  |  | - more than $450 | 10.572 | 0.052 |
|  |  | Family type (vs. Extra) |  |  |
|  |  | - only father | 16.587 | 0.083 |
|  |  | - only mother | 11.778 | 0.085 |
|  |  | - Parents and grandparents living together | 11.111 | 0.104 |
|  |  | - Parents living together | **15.246** | **0.007** |
|  |  | School (vs. Mirero) | **-6.705** | **0.017** |
|  | PRMPCD | Age | **3.535** | **<0.001** |
|  |  | Gender (vs Male) | 0.000 | 1.000 |
|  |  | Digital device exposure (vs. non-exposure group) | 3.883 | 0.119 |
|  |  | Education expense per child (vs less than $15) |  |  |
|  |  | - $15–30 | **-5.389** | **0.030** |
|  |  | - more than $30 | 3.122 | 0.386 |
|  |  | Family income (vs. less than $150) |  |  |
|  |  | - $150–250 | 2.497 | 0.365 |
|  |  | - $250–350 | 5.715 | 0.073 |
|  |  | - $350–450 | -5.824 | 0.288 |
|  |  | - more than $450 | **10.000** | **0.025** |
|  |  | Family type (vs. Extra) |  |  |
|  |  | - only father | 10.000 | 0.206 |
|  |  | - only mother | 3.999 | 0.478 |
|  |  | - Parents and grandparents living together | 2.333 | 0.679 |
|  |  | - Parents living together | 7.026 | 0.132 |
|  |  | School (vs. Mirero) | -3.585 | 0.125 |
|  | SSPFSL | Age | **0.226** | **<0.001** |
|  |  | Gender (vs Male) | 0.072 | 0.654 |
|  |  | Digital device exposure (vs. non-exposure group) | 0.001 | 0.996 |
|  |  | Education expense per child (vs less than $15) |  |  |
|  |  | - $15–30 | -0.297 | 0.086 |
|  |  | - more than $30 | 0.208 | 0.410 |
|  |  | Family income (vs. less than $150) |  |  |
|  |  | - $150–250 | -0.147 | 0.448 |
|  |  | - $250–350 | -0.028 | 0.899 |
|  |  | - $350–450 | 0.179 | 0.644 |
|  |  | - more than $450 | -0.079 | 0.801 |
|  |  | Family type (vs. Extra) |  |  |
|  |  | - only father | -0.286 | 0.608 |
|  |  | - only mother | -0.120 | 0.762 |
|  |  | - Parents and grandparents living together | -0.240 | 0.545 |
|  |  | - Parents living together | -0.120 | 0.714 |
|  |  | School (vs. Mirero) | -0.234 | 0.15 |
| Executive function | SWMBE468 | Age | -0.423 | 0.115 |
|  |  | Gender (vs Male) | **1.509** | **0.048** |
|  |  | Digital device exposure (vs. non-exposure group) | -0.990 | 0.229 |
|  |  | Education expense per child (vs less than $15) |  |  |
|  |  | - $15–30 | -0.606 | 0.461 |
|  |  | - more than $30 | **-2.628** | **0.029** |
|  |  | Family income (vs. less than $150) |  |  |
|  |  | - $150–250 | 0.392 | 0.669 |
|  |  | - $250–350 | -1.423 | 0.180 |
|  |  | - $350–450 | 2.002 | 0.274 |
|  |  | - more than $450 | -1.725 | 0.244 |
|  |  | Family type (vs. Extra) |  |  |
|  |  | - only father | -1.362 | 0.607 |
|  |  | - only mother | 0.907 | 0.631 |
|  |  | - Parents and grandparents living together | 1.587 | 0.401 |
|  |  | - Parents living together | 0.427 | 0.784 |
|  |  | School (vs. Mirero) | 0.023 | 0.976 |
|  | SWMBE4 | Age | 0.112 | 0.087 |
|  |  | Gender (vs Male) | 0.194 | 0.299 |
|  |  | Digital device exposure (vs. non-exposure group) | **-0.473** | **0.018** |
|  |  | Education expense per child (vs less than $15) |  |  |
|  |  | - $15–30 | **-0.504** | **0.012** |
|  |  | - more than $30 | -0.286 | 0.327 |
|  |  | Family income (vs. less than $150) |  |  |
|  |  | - $150–250 | 0.114 | 0.615 |
|  |  | - $250–350 | -0.038 | 0.884 |
|  |  | - $350–450 | -0.036 | 0.936 |
|  |  | - more than $450 | 0.111 | 0.761 |
|  |  | Family type (vs. Extra) |  |  |
|  |  | - only father | -0.124 | 0.846 |
|  |  | - only mother | 0.013 | 0.977 |
|  |  | - Parents and grandparents living together | -0.627 | 0.169 |
|  |  | - Parents living together | -0.355 | 0.346 |
|  |  | School (vs. Mirero) | **-0.574** | **0.002** |
|  | SWMBE6 | Age | -0.162 | 0.293 |
|  |  | Gender (vs. male) | 0.192 | 0.663 |
|  |  | Digital device exposure (vs. non-exposure group) | -0.097 | 0.837 |
|  |  | Education expense per child (vs less than $15) |  |  |
|  |  | - $15–30 | 0.367 | 0.440 |
|  |  | - more than $30 | -0.858 | 0.216 |
|  |  | Family income (vs. less than $150) |  |  |
|  |  | - $150–250 | -0.031 | 0.954 |
|  |  | - $250–350 | -0.770 | 0.211 |
|  |  | - $350–450 | 0.167 | 0.875 |
|  |  | - more than $450 | -0.338 | 0.693 |
|  |  | Family type (vs. Extra) |  |  |
|  |  | - only father | -2.010 | 0.183 |
|  |  | - only mother | 0.053 | 0.960 |
|  |  | - Parents and grandparents living together | 1.533 | 0.155 |
|  |  | - Parents living together | 0.557 | 0.531 |
|  |  | School (vs. Mirero) | 0.147 | 0.742 |
|  | SWMBE8 | Age | **-0.372** | **0.047** |
|  |  | Gender (vs. male) | **1.124** | **0.035** |
|  |  | Digital device exposure (vs. non-exposure group) | -0.420 | 0.467 |
|  |  | Education expense per child (vs less than $15) |  |  |
|  |  | - $15–30 | -0.470 | 0.416 |
|  |  | - more than $30 | -1.485 | 0.079 |
|  |  | Family income (vs. less than $150) |  |  |
|  |  | - $150–250 | 0.310 | 0.631 |
|  |  | - $250–350 | -0.615 | 0.408 |
|  |  | - $350–450 | 1.871 | 0.145 |
|  |  | - more than $450 | -1.498 | 0.149 |
|  |  | Family type (vs. Extra) |  |  |
|  |  | - only father | 0.771 | 0.679 |
|  |  | - only mother | 0.840 | 0.527 |
|  |  | - Parents and grandparents living together | 0.680 | 0.609 |
|  |  | - Parents living together | 0.225 | 0.838 |
|  |  | School (vs. Mirero) | 0.450 | 0.407 |
|  | SWMS | Age | **-5.086** | **<0.001** |
|  |  | Gender (vs. male) | -1.037 | 0.769 |
|  |  | Digital device exposure (vs. non-exposure group) | **13.423** | **0.000** |
|  |  | Education expense per child (vs less than $15) |  |  |
|  |  | - $15–30 | -2.034 | 0.596 |
|  |  | - more than $30 | -0.752 | 0.893 |
|  |  | Family income (vs. less than $150) |  |  |
|  |  | - $150–250 | **-9.464** | **0.027** |
|  |  | - $250–350 | -1.479 | 0.763 |
|  |  | - $350–450 | -6.306 | 0.456 |
|  |  | - more than $450 | -1.204 | 0.860 |
|  |  | Family type (vs. Extra) |  |  |
|  |  | - Only father | 0.331 | 0.978 |
|  |  | - Only mother | **-17.289** | **0.047** |
|  |  | - Parents and grandparents living together | -4.302 | 0.619 |
|  |  | - Parents living together | -7.994 | 0.265 |
|  |  | School (vs. Mirero) | **32.062** | **<0.001** |

MOTML: mean latency from stimulus; RTIFMDRT: median five-choice reaction time; RTIFMDMT: median five-choice movement time; PRMPCI: percent correct immediate; PRMPCD: percent correct delayed; SSPFSL: longest successful sequence; SWMBE468: total between errors; SWMBE4: between errors 4 boxes; SWMBE6: between errors 6 boxes; SWMBE8: between errors 8 boxes; SWMS: strategy score.

Statistically significant data are shown in bold
